# Applications of spatially resolved omics in the field of endocrine tumors

**DOI:** 10.3389/fendo.2022.993081

**Published:** 2023-01-10

**Authors:** Yinuo Hou, Yan Gao, Shudi Guo, Zhibin Zhang, Ruibing Chen, Xiangyang Zhang

**Affiliations:** ^1^School of Pharmaceutical Science and Technology, Tianjin University, Tianjin, China; ^2^General Surgery, Tianjin First Center Hospital, Tianjin, China

**Keywords:** endocrine tumors, liquid chromatography-mass spectrometry, mass spectrometry imaging, microextraction, multi-omics, spatially resolved microproteomics

## Abstract

Endocrine tumors derive from endocrine cells with high heterogeneity in function, structure and embryology, and are characteristic of a marked diversity and tissue heterogeneity. There are still challenges in analyzing the molecular alternations within the heterogeneous microenvironment for endocrine tumors. Recently, several proteomic, lipidomic and metabolomic platforms have been applied to the analysis of endocrine tumors to explore the cellular and molecular mechanisms of tumor genesis, progression and metastasis. In this review, we provide a comprehensive overview of spatially resolved proteomics, lipidomics and metabolomics guided by mass spectrometry imaging and spatially resolved microproteomics directed by microextraction and tandem mass spectrometry. In this regard, we will discuss different mass spectrometry imaging techniques, including secondary ion mass spectrometry, matrix-assisted laser desorption/ionization and desorption electrospray ionization. Additionally, we will highlight microextraction approaches such as laser capture microdissection and liquid microjunction extraction. With these methods, proteins can be extracted precisely from specific regions of the endocrine tumor. Finally, we compare applications of proteomic, lipidomic and metabolomic platforms in the field of endocrine tumors and outline their potentials in elucidating cellular and molecular processes involved in endocrine tumors.

## 1 Introduction

The endocrine system comprises thyroid gland, pituitary gland, parathyroid glands, adrenal glands, pancreas, gonads, pineal gland and thymus. The endocrine glands secrete hormones, which directly enter the bloodstream and come into effect until they reach their target organs. These hormones trigger complicated biological processes, including energy homeostasis, metabolism, reproduction, growth and motions (1).

Endocrine tumors derive from endocrine cells with high heterogeneity in function, structure and embryology, and are characteristic of a marked diversity and tissue heterogeneity (2). They occur in any of the major endocrine organs, including thyroid gland, pituitary gland, parathyroid glands, adrenal glands and the endocrine pancreas (3–6). According to the latest WHO classification, endocrine tumors include pituitary tumors, thyroid neoplasms, parathyroid tumors, paragangliomas and pheochromocytomas, neuroendocrine neoplasms, adrenal cortical tumors and familial endocrine tumor syndromes (7–13). Though most endocrine tumors are benign or low-grade cancers that grow and spread slowly, a few are malignant. For example, thyroid carcinoma is the most common endocrine malignancy (14–17). Based on the GLOBOCAN estimation on cancer incidence and mortality, provided by the International Agency for Research on Cancer, the global incidence of thyroid carcinoma ranked 7th in both sexes and 4th for women in 2020. The mortality of thyroid carcinoma is relatively lower compared to other cancers (0.5 per 100,000 in women and 0.3 per 100,000 in men) (18). The diagnosis of endocrine tumors can be performed by blood/urine tests, ultrasound, computed tomography, magnetic resonance imaging, biopsy and so on (19–22). Fine needle aspiration (FNA) biopsy is frequently recommended to diagnose thyroid neoplasms and parathyroid tumors, where a needle is inserted into the nodules or lumps of patients to collect cells. FNA is a simple diagnostic modality. But it is limited in discriminating ambiguous carcinoma subtypes and additional surgical procedures are required to obtain final diagnosis (23, 24).

The occurrence of endocrine tumors often brings about hyper- or hypo- hormone secretion and potentially causes a succession of disorders, such as hypercalcemia, hypertension and hyperthyroidism (25–28). The tumor tissues comprise of various cell types (such as neoplastic cells, endothelial cells, immune cells, etc.), subpopulations and substructures, which in turn lead to the formation of heterogeneous tissue microenvironment (29–31). Treatments should not only be directed at tumor cells but also should take molecular and cellular interactions within the tumor microenvironment into consideration. High heterogeneity of endocrine tumors is one challenge for the analyses at molecular level. To comprehensively clarify the molecule alternations, both chemical information and spatial distribution of molecules within the tumor microenvironment need to be taken into account. Spatial omics offers increasing insights into pathobiological processes of tumor microenvironment, which allows to understand the location of a cell within tissue, indicates where proteins, lipids or metabolites are expressed in a spatial context and facilitates the identification of unknown cellular regulation processes (32). Mass spectrometry (MS) has shown its advantages in analyzing biomolecules (proteins, peptides, lipids, metabolites, etc.) of complex biological samples at the spatially resolved level (33–35).

Mass spectrometry is an incredibly sensitive analytical technique (down to fmol) that measures the mass-to-charge ratio (*m/z*) of molecules and atoms to determine their molecular weight, enabling qualitative and quantitative analysis for the samples (36, 37). The ion source, mass analyzer and detector are essential components for a mass spectrometer. The sample is first ionized by the ion source to generate a mixture of ions. In the following, the mass analyzer takes the ions and separates them based on *m/z* value. Finally, the ions reach the detector and yield signals. Different ionization techniques include electron ionization, chemical ionization, secondary ion mass spectrometry (SIMS), desorption electrospray ionization (DESI), field ionization, fast atom bombardment, laser desorption/ionization (LDI), electrospray ionization (ESI), matrix-assisted laser desorption/ionization (MALDI) and so on (38). There are also multiple types of mass analyzers, such as time-of-flight (TOF), magnetic sector, linear quadrupole, linear quadrupole ion trap, quadrupole ion trap, Fourier transform-ion cyclotron resonance (FT-ICR) and Orbitrap (39). TOF mass analyzers separate ions according to their *m/z* values based on the length of time it takes them to travel through a flight tube. One advantage of TOF is that it can possess a wide range of *m/z* values. FT-ICR mass analyzers separate ions based on a magnetic field while Orbitrap mass analyzers use an electrostatic field. Both FT-ICR and Orbitrap mass analyzers have high mass resolution and mass accuracy (40, 41). Tandem mass spectrometer (MS/MS) is involved with more than one mass analyzer in a single instrument. In MS/MS, the precursor ions (generated by DESI, ESI, MALDI, etc.) with a specific *m/z* value are selected and fragmented in a collision cell or chamber to generate product ions for detection (42). Fragmentation techniques include collision induced dissociation, high-energy collision dissociation, electron-capture dissociation, electron transfer dissociation, ultraviolet photodissociation and so on (43–47). Mass spectrometry imaging (MSI) is an imaging technique for *in situ* analysis of tissues and cells by determining the relative abundance and distribution of biomolecules (e.g., peptides, proteins, lipids, and metabolites) based on MS (48). For MSI, the sample is ionized pixel by pixel and a mass spectrum is generated for each pixel. The mass spectra are collected at discrete x, y coordinates. For a given *m/z* value, a heat map image can be created by plotting its intensities in all pixels across the scanned area (49–51). MSI can detect and image thousands of biomolecules in a single run, serving as a promising technique in biological and clinical analysis (52–55). Liquid chromatography-mass spectrometry (LC-MS) and liquid chromatography with tandem mass spectrometry (LC-MS/MS) involve the chromatographic separation of analytes followed by the detection of their *m/z* value. With the help of high-performance liquid chromatography or ultra-performance liquid chromatography, the complexity of analytes extracted from the biological samples is effectively reduced and more analytes can be detected by MS (56). To provide a broad coverage of molecules with different chemical and physical properties, different chromatographic platforms are developed, including reversed-phase chromatography, hydrophobic-interaction chromatography and ion exchange chromatography (57–59). LC-MS and LC-MS/MS are widely used in the biological and clinical research, including the field of endocrine tumors (60–65). Spatially resolved LC-MS can be achieved by coupling with laser microdissection (LMD) or liquid microjunction (LMJ), which are two microextraction methods used to extract analytes within the target area of the heterogeneous tumors (66, 67). The combination of microextraction and LC-MS allows the measurement of *m/z* value and spatial location of analytes in the samples.

With the development of MS techniques, chromatographic separation methods and microextraction methods, great progress has been made in clarifying the cellular and molecular mechanisms of endocrine tumorigenesis, progression and metagenesis (68–70). Many biomolecules, such as proteins, lipids and metabolites that present significantly different expression between the tumor tissue and the normal tissue have the potential to act as diagnostic and prognostic biomarkers and therapeutic targets for endocrine tumors (71–73). For example, Coelho et al. reviewed the capability of MS in the diagnosis of thyroid carcinoma from metabolomics. Rossi et al. summarized the potential of steroid profiling by MS in the management of adrenocortical carcinoma (ACC), and Li et al. reviewed the use of MS in proteome-centered multi-omics of human pituitary adenomas (74–76). In this review, we will focus on the application of MS in the field of spatial multi-omics (proteomics, lipidomics and metabolomics) of endocrine tumors, highlighting MSI, LC-MS and microextraction methods. In **Supplementary Table 1**, spatially resolved proteomics, lipidomics, and metabolomics on endocrine tumors are summarized.

## 2 Mass spectrometry imaging in proteomics, lipidomics and metabolomics of endocrine tumors

### 2.1 Mass spectrometry imaging

Mass spectrometry imaging is capable of mapping thousands of biomolecules *in situ* without labelling. Different ion sources and instrument configurations provide different MSI approaches. Secondary ion mass spectrometry-mass spectrometry imaging (SIMS-MSI), matrix-assisted laser desorption/ionization-mass spectrometry imaging (MALDI-MSI) and desorption electrospray ionization-mass spectrometry imaging (DESI-MSI) are the most widely used platforms. SIMS was the first technique employed for tissue imaging (77, 78). The spot size of primary ion beam can be focused to ~50 nm. SIMS is characteristic of high spatial resolution (79–81). In 1997, Caprioli et al. introduced MALDI-MS for tissue imaging (82). With the broad molecule detection coverage, MALDI-MSI is popularly used in the imaging of proteins, lipids and metabolites within biological tissues (83–85). DESI was presented in 2004 and the potential for spatial analysis of plant or animal tissues was demonstrated (86). In 2005, Wiseman et al. reported the first application of DESI-MSI in imaging mouse pancreas, rat brain and metastatic human liver adenocarcinoma tissues (87). These three MSI techniques give full play to their individual advantages in biological and clinical research involved with endocrine tumors (88–93). Their respective advantages and disadvantages are listed in **Table 1**.

**Table 1:** Advantages and disadvantages of SIMS-MSI, MALDI-MSI and DESI-MSI.

MSI methods	SIMS-MSI	MALDI-MSI	DESI-MSI
Ionization method	SIMS	MALDI	DESI
Ionization condition	Vacuum	Vacuum/Ambient	Ambient
Spatial resolution	Down to 50-100 nm	Down to ~ 1 μm	Down to ~ 10 μm
Advantages	High spatial resolution	High applicability to biomolecules Fast acquisition with up to 40 pixel per second	Minimum sample preparation
Disadvantages	Abundant fragmentation Less reproducible Expensive	Require matrix application	Low spatial resolution

#### 2.1.1 Secondary ion mass spectrometry-mass spectrometry imaging

**Figure 1:**
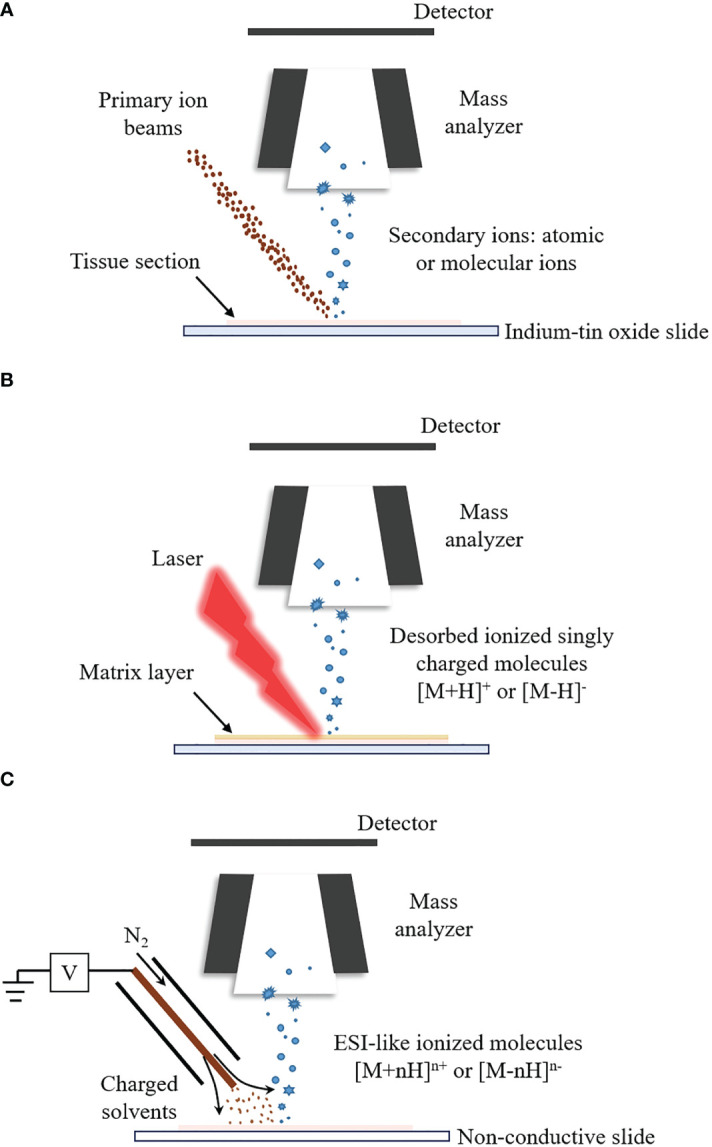
The ionization principles for SIMS-MSI, MALDI-MSI and DESI-MSI. A mass spectrometer is at least composed of ion source, mass analyzer and detector. Different ion sources determine different ionization process. **(A)** Ionization process of SIMS-MSI. A primary ion beam possessing energy strikes the sample surface, causing the interaction between the ions and the surface. The interaction processes bring about the emission of atoms and molecules from the sample surface. **(B)** Ionization process of MALDI-MSI. Before the analysis, the matrix is applied to the sample surface. The matrix forms co-crystals with the analytes. The co-crystals can absorb the laser’s energy upon laser irradiation. The energy uptake then causes evaporation and desorption/ionization of the analytes. **(C)** Ionization process of DESI-MSI. It is carried out by applying pneumatically-assisted electrospray, which produces charged solvent droplets directly at the sample surface. The charged droplets impact the surface and produce gaseous ions.

SIMS-MSI can reach micron and submicron spatial resolution, capable of imaging single cells or subcellular organelles (94–96). The highest spatial resolution of SIMS-MSI, down to tens of nanometers, outperforms the other two MSI techniques (97–99). The principle of SIMS-MSI is shown in **Figure 1A**. In SIMS-MSI, a high-energy primary ion beam strikes the sample surface, causing the interaction of sputtering, ion reflection and recoil sputtering between the ions and the surface. The interaction processes result in the emission of secondary ions (100).

There are various types of commercially available primary ion beams, including monatomic (Au^+^, Cs^+^ and O^-^) and polyatomic ion beams ( ${\text{C}}_{60}^{+}$ ), liquid metal ion guns (LMIGs) ( ${\text{Bi}}_{3}^{+}$ and ${\text{Au}}_{3}^{+}$ ) and gas cluster ion beams (GCIBs) ( ${\text{Ar}}_{4000}^{+}$ , (CO_2_ $({\text{CO}}_{2}{)}_{2000}^{+}$ and $\left({\text{H}}_{2}\text{O}{)}_{2000}^{+}\right)$ (101–106). The sensitivity and spatial resolution of SIMS-MSI are influenced by the type, the energy and the focusing spot size of the primary ion beams. The monatomic ion beams limit SIMS-MSI to the detection of elements or very small (e.g., diatomic) fragments of molecules (107). LMIGs produce increased sensitivity while still being readily focused to tens to hundreds of nanometers. The use of polyatomic ion beams and GCIBs further improves the sensitivity to higher mass species (102). Lipids and metabolites have been spatially resolved in different cell types of breast cancer (105). A primary ion beam with high energy tends to have high secondary ion yields. However, highly energetic primary ion beams induce strong fragmentation of the analytes and generate very small ion fragments (97). The spatial resolution of SIMS-MSI can reach a few hundred nanometers when the spot size of primary ion beam is focused ≤ 50 nm (108). For GCIBs, the typical optimal spot size is around 1-5 μm and the increased sensitivity is obtained at a loss of spatial resolution (107). For SIMS-MSI, it is essential to select a primary ion beam with appropriate energy (typically between 25 and 70 keV kinetic energy) and focusing spot size to obtain ideal sensitivity and spatial resolution. The selection depends on the types of target tissues and target analytes (109).

#### 2.1.2 Matrix-assisted laser desorption/ionization-mass spectrometry imaging

MALDI-MSI can measure molecules with a wide mass coverage, ranging from 100 Da to beyond 100kDa; and it can measure molecules with different polarities, ranging polar lipids to ionic metabolites (110–112). The majority of MSI studies are performed by MALDI-MSI (113). In MALDI-MSI, the desorption/ionization of the analytes is performed with the assistance of matrices, as described in **Figure 1B** (114). The matrix is applied to the sample surface and form co-crystals with the analytes. The co-crystals can absorb energy upon laser irradiation. The energy uptake then causes evaporation and ionization of the analyte (82, 115).

The matrices do make a great difference to the ionization process and the selection mainly depends on chemical properties of the molecules of interest (116). The matrices are generally crystalline solids of low vapor pressure. Including 2,5-dihydroxybenzoic acid (2,5-DHB), alpha-cyano-4-hydroxycinnamic acid (CHCA), sinapinic acid (SA), 9-aminoacridine (9-AA) and 1,5-diaminonaphthalene, a diversity of common organic matrices that fit the ionization of different classes of molecular species are listed in **Table 2** (129, 132–138). SA is frequently used to assist the ionization of intact proteins (139). 2,5-DHB can be used to image lipids, peptides, and amino acids in the positive ion mode (140–142). 9-AA is preferred to be performed under negative ion mode for the ionization of polar metabolites (143). The application of matrices is required to assist the ionization and subsequent detection of analytes. However, matrices sometimes bring about ion suppression effects and induce sensitivity loss for analytes (144). CHCA is commonly used as a MALDI matrix in the ionization of peptides. When the peptide sample is quite dilute and/or the sample contains salts, the CHCA matrix can form clusters with *m/z* value above 500 (145, 146). These intense CHCA clusters may interfere with peptide signals and complicate the spectra. To reduce the ion suppression effects of CHCA matrix, Ucal et al. used ammonium phosphate monobasic as the addictive of CHCA in the analysis of thyroid carcinoma tissue and found that the addition of ammonium phosphate monobasic could decrease CHCA cluster formation and improve the peptide signals (147). Schlosser et al. utilized different matrix components, additives and a cationizing agent to analyze the effects of matrix composition on signal suppression and found that the mixture of 2,5-DHB and CHCA yielded highly improved ion signals in peptide analysis, compared with using CHCA alone (148). Apart from the matrix clusters, matrices (such as CHCA and 2,5-DHB) could also form adducts with lipids, amines and amino acids. The metabolite-matrix adducts decrease the intensities of the metabolites and further complicate the MS spectra (149).

**Table 2:** Common organic matrixes applied in MALDI-MSI.

Common organic matrixes in MALDI-MSI	Target molecules
SA (117, 118)	Proteins
2,5-dihydroxyacetophenone (119)	Proteins
CHCA (112, 120)	Peptides and lipids
2,5-DHB (121–123)	Lipids, peptides and drugs
9-AA (124)	Lipids and metabolites
1,5-diaminonaphthalene (125, 126)	Lipids and metabolites
1,8-bis (dimethylamino) naphthalene (127)	Lipids and metabolites
Graphene oxide (128)	Lipids
Hydralazine (129)	Proteins, lipids and metabolites
N‐ (1‐naphthyl) ethylenediamine dihydrochloride (130)	Lipids and metabolites
Norharman (131)	Lipids
Quercetin (132)	Lipids

#### 2.1.3 Desorption electrospray ionization-mass spectrometry imaging

DESI-MSI is carried out by applying pneumatically-assisted electrospray to produce charged solvent droplets directly at the sample surface (150, 151). The charged droplets impact the surface and produce gaseous ions, which are mainly multiply charged as in the case with ESI (**Figure 1C**) (86, 152–154). DESI-MSI is performed under ambient conditions and requires no matrix application or other advanced sample preparation, allowing biological tissues to be analyzed in their native state (155, 156). DESI-MSI is one of the MSI methods that have the least destructive effect on the biological tissues. The same tissue section is able to be analyzed repeatedly (77, 157, 158). The spatial resolution of DESI-MSI is typically 150-200 μm and the maximum is better than 10 μm (159).

Before a DESI-MSI analysis, it is essential to optimize the following parameters, including the spray solvent composition, the velocity of the spray gas, the spray-to-surface and sampling orifice-to-surface distance, sprayer-to-surface angle and surface-to-desolvation capillary angle (160–165). Failure of optimization of DESI-MSI parameters will lead to poor sensitivity and/or low spatial resolution.

### 2.2 Sample preparation for mass spectrometry imaging

Sample preparation protocols are of great importance for the MSI analysis, mainly encompassing sample collection, processing and post-sectioning treatments (166, 167). Improper sample collection and storage may induce degradation of the analytes or introduce interferences, such as blood and chemical reagents (168–170). Non-standard operations may cause variations in sensitivity, spatial resolution and mass accuracy among technical replicates, leading to poor reproducibility (171). And the obtained MSI results cannot reflect the real nature of tissues under study (172). Therefore, it is essential to standardize each sample preparation protocol before MS data acquisition (173).

#### 2.2.1 Fresh frozen tissues, formalin fixed paraffin embedded tissue blocks and cytologic samples

As distinguished from LC-MS, MSI maps biomolecules directly from the tissue surface *in situ*. Therefore, it is important to preserve the original nature and integrity of the tissue during the process of sample preparation and data acquisition (51, 116). Most samples of endocrine tumors used in MSI analysis are surgically resected suspicious nodules or lumps, e.g., fresh frozen tissues and formalin fixed paraffin embedded (FFPE) blocks of tissues. In some cases, cytological samples are obtained by FNA (117, 174, 175). For fresh frozen tissues or cytological samples, it is necessary to reduce the time spent in harvesting the sample and the acquired fresh sample needs to be snap-frozen in liquid nitrogen right away (176–178). At last, samples can be preserved below -80° until the analysis. Heat-stabilization and *in situ* focused microwave irradiation are two alternatives to snap freezing the freshly harvested tissues (179). Heat-stabilization inactivates the enzymes by quickly heating the tissues to 95°C while *in situ* focused microwave irradiation heats the tissues with focused microwaves to deactivate enzymes within 2 seconds (180, 181). The processed tissues can be preserved in the freezer extended periods.

For FFPE tissue blocks, the fixation can preserve the cellular architecture and the composition of cells in the tissue; however, it also results in cross-linking between nucleic acids, between proteins and between nucleic acids and proteins (182). The cross-linking between proteins inhibits the proteomic analysis by MS seriously (183). This challenge can be overcome by specific sample processing and post-sectioning treatments.

#### 2.2.2 Sample processing

**Figure 2:**
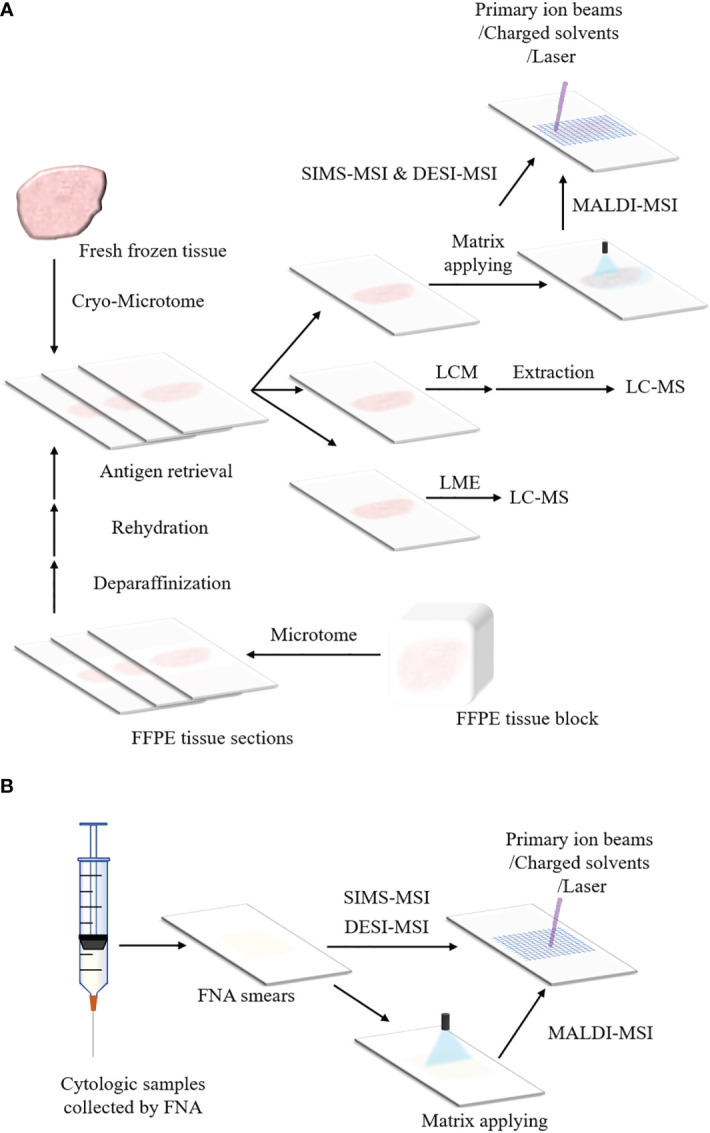
Schemes of sample preparation for MSI and microproteomics. **(A)** Preparation protocols of fresh frozen tissues and FFPE tissue blocks for MSI and LMD or LMJ guided microproteomics. The fresh frozen tissue is sliced into sections by cryo-microtome and the tissue sections are placed on ITO slides or non-conductive slides. Then the tissue sections can be processed with MSI (For MALDI-MSI, matrix applying before data acquisition is necessary). The tissue section can also be processed with LMD or LMJ. For LMD, the region of interest within the tissue section is cut off and extracted, followed by LC-MS. For LMJ, the extracts obtained from the target region within the tissue surface can be directly analyzed by LC-MS. After data acquisition, data analysis is performed. For the FFPE tissue block, it is sliced into tissue sections by microtome. These FFPE tissue sections can be analyzed by MSI or LC-MS until they are treated with deparaffinization, rehydration and antigen retrieval. **(B)** Preparation protocols of cytologic samples for MSI. The cytologic samples are collected by FNA. The cytologic samples are smeared onto the ITO slides or non-conductive slides for the analysis of MSI.

**Figure 2A** shows sample preparation protocols of fresh frozen tissues in MSI. The first step is to slice the tissue into thin sections by cryo-microtome (typically 3-20 μm) (184–186). The tissue sections are then mounted onto the glass slides, metal targets or indium-tin oxide (ITO) coated glass slides. Before sectioning fresh frozen tissues, various embedding media can be used to preserve the morphology of the tissues and assist with the tissue section, including optimal cutting temperature medium (OCT), carboxymethyl cellulose, gelatin, agarose or ice (121, 187–191). However, OCT could suppress analyte signals and is not recommended in MSI (189, 192). A universal embedding media composed of hydroxypropyl methylcellulose and polyvinylpyrrolidone has been demonstrated to be compatible with SIMS-MSI, MALDI-MSI and DESI-MSI (193). The section temperature significantly varies according to tissue types (172, 194). Tissues containing water are sectioned at higher temperature whereas tissue samples that contain more fat can be sectioned at a lower temperature (195). For FFPE tissue blocks, the tissue sections can be analyzed by MSI after a series of treatments including deparaffinization, rehydration and antigen retrieval, as presented in **Figure 2A**. In addition, the cytologic samples collected by FNA are smeared onto ITO slides or non-conductive slides for the following MSI analysis (**Figure 2B**) (118, 174).

Histology staining is frequently cooperated with MSI to connect the histology features of the tissue with the molecular profiles (196). It has been demonstrated the distribution of biomolecules obtained by MSI correlates well with the histology structure of the tissue (197–199).

#### 2.2.3 Post-sectioning treatments

Post-sectioning treatments aim to enhance the sensitivity of analytes of interest. Biological tissues contain numerous molecular species, whose abundance varies widely. If the abundance of targeted analytes is relatively low, it is necessary to tailor post-sectioning treatments (200).

Washing is a common post-sectioning treatment, aiming to remove those interfering molecules and increase the signal intensity of target analytes within the samples (201). The washing strategy with ethanol solutions and water has been commonly applied in the proteomic analysis to remove interfering salts or lipids (202, 203). The washing solution with ammonium formate or ammonium acetate was proved to enhance detection sensitivity of lipid species (204).

For bottom-up proteomics, on-tissue digestion is performed, involving proteolytic digestion of proteins before analysis by MS (205–207). It is especially advantageous for FFPE samples. On-tissue digestion applies trypsin solution to the sample surface after antigen retrieval. The trypsin facilitates the digestion of cross-linking proteins (136, 208). Abdelmoula et al. studied FFPE tissue blocks of oncocytic follicular thyroid cancer by MALDI-MSI (209). Before the matrix application, the tissue section was proceeded with deparaffinization, dehydration, antigen retrieval and trypsin digestion. The MSI results showed that hundreds of proteolytic peptide ions were detected and that many of them exhibited specific distributions in association with the histological structure of the tissues. FFPE tissues treated with on-tissue digestion is proved to be compatible with the following proteomic analysis (210).

Chemical derivation is another post-sectioning strategy to increase the detection sensitivity of specific lipids and metabolites, such as steroids, catecholamine, and phospholipids (PLs) (211–215). Ibrahim et al. performed an on-tissue chemical derivation of dopamine, epinephrine and norepinephrine with 4- (N-methyl) pyridinium boronic acid in SIMS-MSI and LDI-MSI of porcine adrenal gland tissue (216). They demonstrated that the derivation significantly improved the detection sensitivity of catecholamines in tissue sections for both MSI techniques. Wang et al. achieved simultaneous imaging of free fatty acids (FFAs) and phospholipids with a high sensitivity in thyroid cancer tissue by chemical derivation of FFAs with N,N-dimethylpiperazine iodide (127).

#### 2.2.4 Data processing and analysis

The raw data of MSI is made of individual spectra with spatial and molecular information, so it is generally complex and high dimensional. The basic steps of MSI data processing consist of denoising, baseline subtraction, normalization, peak picking and peak alignment (217, 218). Due to variations in instruments, sample heterogeneity and sample preparation, noises and fluctuations in mass exist in the MSI raw data. Data processing helps reduce the technical and analytical variations, providing a more reliable elaboration of the MSI dataset (219). After data processing, the MSI dataset can be submitted to the statistical analysis. Huang et al. developed a data processing pipeline for spatially resolved metabolomics analysis (219). In the pipeline, 7 data pre-treatment methods (centering, normalization, automatic scaling, UV scaling, Pareto scaling, log transformation and square root transformation) were investigated before a partial least squares discriminant analysis. And the following score test and classification test revealed that log transformations can reveal more low-abundance biomarkers and produce better classification results.

The data analysis for MSI can be performed with MassImager, Biomap, Data Explorer, MALDI Imaging Team Imaging Computing System, FlexImaging, oMALDI Server 5.1 and SCiLS Lab (116, 220–225). Multivariate methods are applied, such as principal component analysis, clustering methods, factorization methods and classification methods (226). These statistical analyses may discriminate differential molecules between normal and tumor tissues and find potential biomarkers for the tumor. Different from the publicly available and commercial software tools, *Cardinal* is an open-source R package that implements data processing (normalization, baseline correction, peak detection and peak alignment), visualization of mass spectra, statistical segmentation (principal component analysis, Spatially-Aware and Spatially Aware Structurally Adaptive) and classification (partial least squares discriminant analysis and orthogonal projections) of ion images for MSI (227). *Cardinal* also introduced Spatial Shrunken Centroids, a novel method for model-based image segmentation and classification.

### 2.3 Mass spectrometry imaging in proteomics of endocrine tumors

Gene alternations play a fundamental role in endocrine tumors (228, 229). For example, BRAF (v-Raf murine sarcoma viral oncogene homolog B1) mutations, RAS (rapidly accelerated fibrosarcoma) mutations and RET (Proto-oncogene tyrosine-protein kinase receptor Ret) rearrangements are common genetic alternations in papillary thyroid carcinoma (PTC) and follicular thyroid carcinoma (FTC); GNAS (guanine nucleotide binding protein, alpha stimulating) gene mutations happens in sporadic pituitary adenomas while MEN1 (menin 1) and AIP (aryl hydrocarbon receptor interacting protein) mutations in family isolated pituitary adenoma (230, 231). Though great achievements have been made in elucidating the mechanism and pathology of endocrine tumors through genomic analysis, the gene expression and protein expression lack apparent correlation (4, 232–234). Proteins are gene products, the executors of cellular processes and more closely related to the phenotypes (235). Proteomics is complementary to genomics in revealing the alternations in structure, function and interactions of proteins in tumorigenesis and tumor progress. With MSI as the analysis tool, proteomics in endocrine tumors has showed the potential to discover different protein signatures between the tumor tissues and adjacent normal tissues and to discriminate among different subtypes of thyroid cancers. The spatially resolved proteomics of endocrine tumors can contributed to a better understanding of the overall mechanism involved in the tumorigenesis, progression, and metastasis.

The application of MSI in proteomics is capable of discriminating between the normal tissue and the cancer tissue as well as distinguishing between different subtypes of thyroid tumors (236–238). Mainini et al. analyzed the cytological smears obtained by *ex vivo* FNA from 7 patients with hyperplastic nodules or PTC using MALDI-MSI (239). The MSI data was processed with hierarchical cluster analysis and principal component analysis to evaluate the different proteomic expressions. And hyperplastic nodules and PTC were successfully discriminated by hierarchical cluster analysis and principal component analysis. Pagni et al. used MALDI-MSI to compare the protein profiles of cytologic samples obtained from patients diagnosed as hyperplastic nodules, Hürthle cell follicular adenoma, medullary thyroid carcinoma (MTC) and PTC (176). They evaluated 6 proteins whose expression in PTC were different from that of benign lesions, but similar to that of MTC. Different protein profiles that could distinguish between PTC and MCT were also detected. Calligaris et al. presented the application of MALDI-MSI in detecting and discriminating nonpathological human pituitary glands, hormone secreting and non-secreting human pituitary adenomas (240). They validated the capability of MALDI-MSI to image prolactin (PRL), growth hormone (GH), adrenocorticotropic hormone (ACTH) and thyroid stimulating hormone (TSH) within normal glands and adenomas, but also submitted the MSI data to principal component analysis to evaluate the different protein signatures among nonpathological human pituitary glands, hormone secreting and non-secreting human pituitary adenomas. It was revealed that the sensitivity and specificity of MSI data distinguishing ACTH secreting adenomas from nonpathological pituitary were 100% and 93%, the sensitivity and specificity of MSI data distinguishing GH secreting adenomas from nonpathological pituitary were 82% and 100% and the sensitivity and specificity of MSI data distinguishing PRL secreting adenomas from nonpathological pituitary were 50% and 100%, respectively.

The application of MSI proteomics is also capable of finding potential protein biomarkers for the diagnosis of endocrine tumors (241). Nipp et al. performed MALDI-MSI and immunohistochemistry (IHC) on PTC tissues to find biomarkers for the metastasis of PTC (242). Using MALDI-MSI, they successfully found that thioredoxin, S100-A10 (p11, the ligand of Annexin-II) and S100-A6 (Calcyclin) could specially distinguish metastatic PTC from non-metastatic PTC. And IHC validated that these three overexpressed proteins were significantly associated with lymph node metastasis of PTC with *p* values < 0.005 (*p* value for thioredoxin: 0.00003; *p* value for S100A10: 0.00018; *p* value for S100-A6: 0.0013; Fisher’s exact test).

The application of MSI in proteomics is capable of bringing insight into the endocrine tumor progression. Tissue necrosis is common in advanced and aggressive solid tumors (243). Scott et al. studied the N-linked glycosylation of proteins in human thyroid cancer tissue by MALDI-MSI (244). They demonstrated that proteins with high mannose or branched glycans were specially distributed in the cancer and stromal regions, whereas the glycans of proteins in necrotic regions presented limited branching, contained sialic acid modification and lacked fucose modification. Gawin et al. used MALDI-MSI to compare protein profiles between the primary PTC located in the thyroid gland and the PTC with synchronous metastases in regional lymph nodes (245). Thirty-six proteins were found remarkably different in abundance between primary PTC and metastatic PTC, which were then annotated as proteins involved in the organization of the cytoskeleton and chromatin, as well as proteins involved in immunity-related functions.

### 2.4 Mass spectrometry imaging in lipidomics of endocrine tumors

Lipids are hydrophobic or amphipathic compounds with great differences in their chemical composition and structure (246). Lipids are divided into 8 categories: fatty acyls, glycerolipids, glycerophospholipids, sphingolipids, sterol lipids, prenol lipids, saccharolipids and polyketides (247). Lipids involve in many essential cellular processes, including chemical-energy storage, composition of cell membrane bilayer, cell-cell interactions and cellular signal transduction. Lipidomics has been defined as a tool of full characterization of lipid molecular species and of their biological roles with respect to expression of proteins involved in lipid metabolism and function, including gene regulation (248). Abnormal lipid metabolism has been considered as a key feature of cancers (249–252). Stearoyl-CoA desaturase (SCD1) has been proved to be highly expressed in PTC, FTC and anaplastic thyroid carcinoma (ATC) (253, 254). SCD1, a fatty acyl desaturase encoded by stearoyl-CoA desaturase 1 gene, plays an important role in *de novo* lipid biosynthesis (255). It is a rate-limiting enzyme in the reaction of producing monounsaturated fatty acids (such as oleic acid and palmitoleic acid) from saturated fatty acids (such as stearic and palimitic acid). Monounsaturated fatty acids are the substrates for the synthesis of triglycerides, sphingolipids, glycolipids, (PLs), and other lipoproteins (256, 257). The elevated SCD1 promotes the proliferation, migration and invasion of cancer cells in PTC, FTC and ATC. Several research groups have focused on the lipidomics of endocrine tumors by MSI to analyze multiple lipid species and detect lipid alternations during the tumorigenesis (177, 258).

MSI showed the competency for detecting specific phosphatidylcholine (PC), sphingomyelin (SM) and phosphatidic acid (PA) species that may associate with the pathological behaviors of PTC. Ishikawa et al. investigated the distribution of lipids within cancerous and normal tissues from PTC patients using MALDI-MSI and MS/MS identification (259). The MSI analysis was performed by MALDI-TOF/TOF with 2,5-DHB as the matrix. And it was found that three species with *m/z* value 798.5, 796.5 and 741.5 were remarkably increased in the cancerous tissue compared to the normal tissue. A hybrid quadrupole/TOF mass spectrometer equipped with an orthogonal MALDI source was used to identify these three ions as [PC (16:0/18:1)+K]^+^ and [PC (16:0/18:2)+K]^+^ and [SM (d18:0/16:1)+K]^+^, respectively. Wojakowska et al. employed MALDI-MSI to find lipids that could discriminate between PTC tissues and adjacent non-cancerous thyroid tissues (260). They found that intensities of PC (32:0), PC (32:1), PC (34:1), PC (36:3), SM (34:1), SM (36:1) and PA (36:2) and PA (36:3) of the cancerous tissue were significantly higher than that of the non-cancerous tissue.

MSI is also competent for imaging differential molecular signatures for oncocytic thyroid tumors, e.g., the abnormal expression of cardiolipins (CLs). CLs play an important role in the stability and integrity of mitochondrial structure and function. There is increasing evidence in the CL metabolism reprogramming of cancers. However, the exact mechanism by which CLs modulate cancer remains to be clarified (261). Zhang et al. conducted the DESI-MSI analysis to image and characterize the lipid composition for the oncocytic thyroid tumors (Hurthle cell adenoma and Hurthle cell carcinoma) (262). They found that CL species were distributed in the oncocytic thyroid tumor with an abnormally high abundance and diversity, as compared with the non-oncocytic thyroid tumors (PTC, FTC and follicular adenoma) and normal thyroid samples. Feider et al. applied the integrated DESI-field asymmetric ion mobility spectrometry-MSI approach to measure CLs in oncocytic thyroid tumors (163). They validated the existence of abundant CL species in the entire thyroid tissue section and managed to identify *m/z* values of 723.479, 737.494 and 747.473 as CL (72:6), CL (74:8) and CL (76:9). The ion images of MSI demonstrated that oncocytic thyroid tumor was present throughout the tissue section, MSI images were consistent with histologic images. The spatial distribution of CLs among the entire tissue has the potential to indicate specific locations of oncocytic thyroid tumor.

Moreover, MSI is competent for the detection of FFAs and PLs of the cancer tissue and the para-cancer tissue to elucidate the relatives between changes of FFAs and PLs and the cancer development (263–265). FFAs are an essential constituent of PLs. It has been revealed that FFAs greatly influence the energy storge in the cancer microenvironment and act as second cellular messengers (266). The metabolism of FFAs is an essential step in *de novo* lipogenesis, which is more active in the cancer tissue compared with the normal tissue (267, 268). Wang et al. simultaneously imaged FFAs and PLs in the thyroid cancer tissue and the para-cancer tissue by MALDI-MSI (127). They found that the intensities of 7 FFAs (arachidic acid (C20:0), oleic acid (C18:1), linolenic acid (C18:3), palmitoleic acid (C16:1), arachidonic acid (C20:4), docosahexaenoic acid (C22:6) and linoleic acid (C18:2)) were significantly higher in the cancer tissue than that of the para-cancer tissue. The correlation between FFAs and PLs was analyzed by submitting the intensity of each detected PL and FFA derivative in each spot for the cancer tissue and the para-cancer tissue to Spearman correlation analysis. The heatmaps of the correlation between FFAs and PLs in thyroid cancer samples were created to reveal that the saturated FFAs (C16:0 and C18:0) were positively correlated with PLs. This is because palmitic acid (C16:0) is the main product of *de novo* fatty acid synthesis and a precursor for the synthesis of other fatty acids. Combined with the upregulation of palmitic acid in cancer tissue, this phenomenon is due to the more active *de novo* synthesis of fatty acids in cancer tissue to provide abundant precursors for other lipid metabolism.

### 2.5 Mass spectrometry imaging in metabolomics of endocrine tumors

Metabolites are intermediate end products generated by chemical reactions within cells, tissues and organs (269). Metabolomics, focusing on the altered metabolites and metabolic pathways within the biological sample, is a promising technique in shedding light on the molecular mechanisms of endocrine tumors (270–272). MSI has made great progress in the metabolomic analysis of endocrine tumors, involving detection of altered metabolites, elucidation of tumor metabolism reprogramming and identification of possible biomarkers (273).

MSI has the ability to present the histology heterogeneity but also can reveal the metabolic heterogeneity within the tumor. Huang et al. studied the metabolism of PTC by ambient pressure DESI-MSI (274). They built a spatially resolved metabolomic data processing pipeline that revealed the tumor microregion heterogeneity. A clear discrimination among the tumor, the stromal and the normal tissue was shown. The para-cancer region was further segmented into different microregions based on the differential metabolic profiles. Additionally, this study showed that the abundances of phenylalanine, leucine and tyrosine were the highest in the tumor region, followed by the stromal region, lowest in the normal tissue. It has been revealed that amino acids are involved in glycolysis and tricarboxylic acid cycles, reshaping the cellular metabolism (275). Cancers demand abundant amino acids to promote cancer cell proliferation, invasion and metastasis. Amino acids were usually present to be increasingly expressed in PTC (276–279).

MSI has the ability to help elucidate the molecular mechanism of the pheochromocytoma. The adrenal medulla, in the central part of the adrenal gland, is composed of chromaffin cells that synthesize catecholamines. The hormones exert their effects by acting on alpha- and beta- adrenoreceptors in the central nervous system and the periphery (280). The “fight or flight response” is a key mechanism and causes a number of physiological changes, such as increased blood pressure, increased cardiac output and increased glycogenolysis in liver and muscle tissue (281). Pheochromocytoma is formed in the adrenal medulla. This type of tumor produces and releases a large amount of circulating catecholamines and leads to a constant activation of the “fight or flight response” (282). Takeo et al. visualized the distribution of adrenaline and noradrenaline in the normal tissue and the pheochromocytoma tissue (213). They demonstrated that both catecholamines were distributed in the adrenal medulla of the normal tissue, whereas pheochromocytoma tissue showed a moderate adrenaline level and an elevated level of noradrenaline with a homogeneous distribution among the whole tumor region.

MSI has the potential to provide additional support for the hypothesis that aldosterone-producing cell cluster (APCC) is the origin of aldosterone-producing adenoma (APA) (283). It is reported that aldosterone or 18-oxocortisol is a potential serum marker of APA. Sugiura et al. visualized the distribution of aldosterone or 18-oxocortisol in APCC, possible APCC-to-APA transitional lesions and APA by MALDI-MSI (284). The ion images revealed that aldosterone and 18-oxocortisol congregated within the tumor regions where aldosterone synthase was distributed. The imaging results of possible APCC-to-APA transitional lesions even suggested a path of cellular migration from APCC to form APA inside the adrenal glands. Sun et al. used MALDI-MSI to compare the metabolomic phenotypes between APCC and APA (285). They processed the MALDI spectra by component analysis. Depending on their respective metabolite distribution patterns, the APCC were divided into 2 subgroups. Metabolic profiles of APCC in subgroup 1 were distinct from APA, whereas subgroup 2 displayed metabolic profiles similar to the APA group. Compared to subgroup 1, subgroup 2 presented increased hexose monophosphate shunt, enhanced metabolism of tryptophan *via* the kynurenine pathway and the significant enhancement of N-acetylglucosamine, which may be related to cell proliferation and APCC to APA transition.

MALDI-MSI has the potential to discriminate endocrine tumors with different genotypes based on the metabolic profiles. By using MALDI-FT-ICR with 9-AA matrix, Murakami et al. analyzed the metabolism of APAs by MSI (286). The metabolic data was processed with ortho-PLSDA clustering between KCNJ5- (potassium voltage-gated channel subfamily J member 5) and CACNA1D- (calcium voltage-gated channel subunit alpha1 D) mutated APAs. One hundred and thirty-seven differential metabolites were screened out (adjusted *p* value < 0.05). In the following, the significantly altered metabolites were submitted to the pathway analysis and the activation of purine metabolism in KCNJ5-mutated APAs was demonstrated (pathway impact = 0.13, *p* < 0.001, and FDR < 0.001).

## 3 Spatially resolved microproteomics in endocrine tumors

### 3.1 Spatially resolved microproteomics and microextraction approaches

Conventional proteomics usually performs extraction by preparing the whole piece of tissue into homogenate. The final protein or peptide sample is injected into the LC-MS system in solution. The homogenization process leads to the loss of the histological structure of the tissue and the spatial localization of the analytes (287). Moreover, proteins with low abundance sometimes cannot be detected due to the interference of abundant proteins (288). These challenges can be overcome by spatially resolved microproteomics, which allows quantitative and comparative proteomic analysis within a relatively small surface area (μm) in the tumor microenvironment (61, 289–295). Spatially resolved microproteomics is achieved by the collaboration of LC-MS and microextraction approaches. Laser microdissection (LMD) and liquid microjunction (LMJ) are two general microextraction approaches that help extract proteins from a relatively limited area of the sample surface (296–298).

#### 3.1.1 Laser microdissection

LMD can isolate and harvest subpopulations of tissue cells relying on either infrared (IR) laser or ultraviolet (UV) laser coupled with a microscope (34). The histology structure of the sample is present under the microscope and regions of interest are determined by direct microscopic visualization (299).

**Figure 3:**
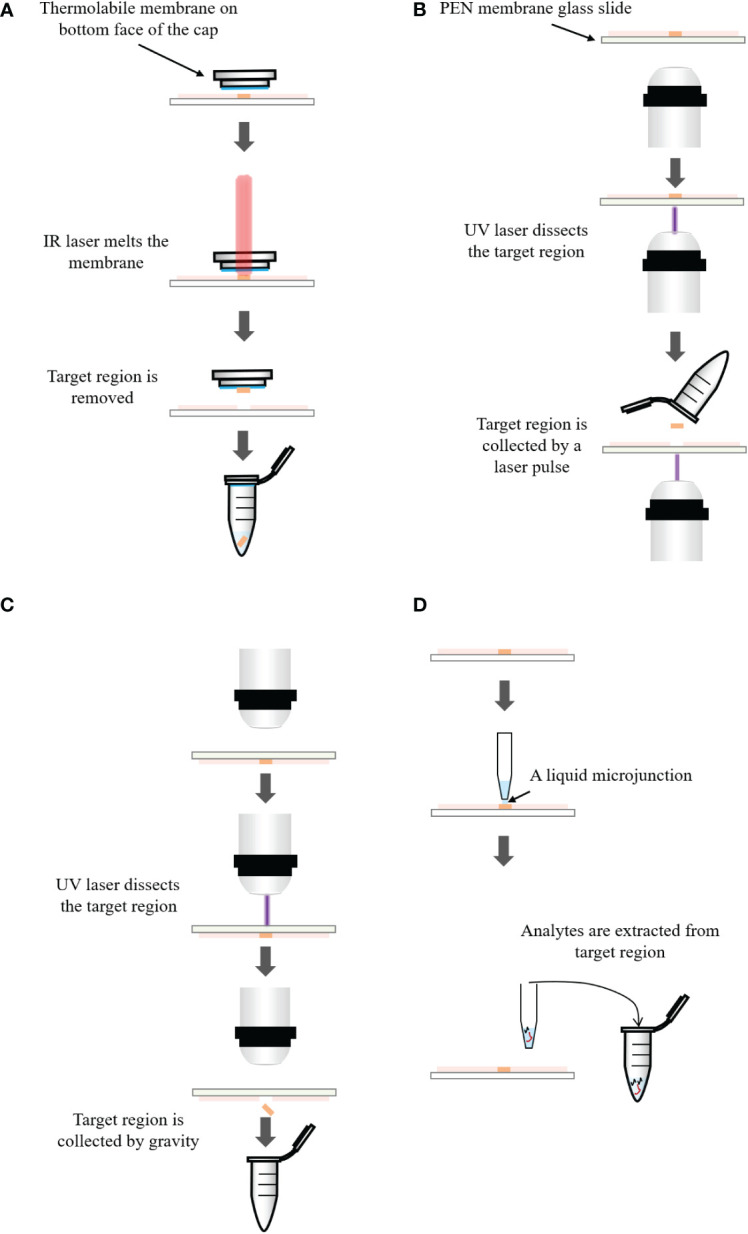
**(A)** Principle of the Arcturus laser capture microdissection. A thermolabile membrane on bottom face of the cap is placed on the tissue section. The infrared (IR) laser activates the membrane which extends to the tissue. The adhesion force of the tissue to the activated membrane exceeds that to the glass slide. The selected area is removed from the tissue. **(B)** Principle of the Zeiss’s PALM microdissection. The tissue section is mounted on a polyethylene napthalate (PEN) membrane coated glass slide. After selecting the region of interest, ultraviolet (UV) laser ablates the surrounding cells and cuts away the selected area, which is then transported into a collection tube by a defined laser pulse against gravity. **(C)** Principle of the Leica LMD microdissection. The tissue section is mounted on the PEN membrane glass slide and placed upside down on the stage. The target tissue is dissected by the laser and directly falls into a collection tube underneath the tissue section. **(D)** Principle of liquid microjunction extraction. The probe aspirates the extraction solvent and dispenses a portion onto the tissue surface to create a liquid microjunction between the probe and the tissue surface. After a predefined extraction time, analytes that are soluble in the solvent are extracted into the liquid microjunction. The extracted solution can be analyzed by LC-MS directly.

**Figures 3A–C** respectively introduce the principles of three LMD systems from different vendors. In the system of Arcturus laser capture microdissection, the tissue section is located on the glass slide. A thermolabile membrane on bottom face of the cap is placed on the tissue section. The IR laser activates the membrane and the melted membrane extends to the tissue. The adhesion force of the selected tissue area to the activated membrane exceeds that to the glass slide. The selected area is removed from the tissue (300). In Zeiss’s PALM microdissection, the tissue section is mounted on a polyethylene napthalate (PEN) membrane glass slide. After selecting the region of interest, the UV laser ablates the surrounding cells and cuts away the selected area (301). The cut-off areas are transported into a collective tube by a defined laser pulse against gravity. In the Leica LMD microdissection, the tissue section is mounted on the PEN membrane glass slide and placed upside down on the stage. The target tissue is dissected by the UV laser and directly falls into a vessel underneath the tissue section by gravity (302).

#### 3.1.2 Liquid microjunction

LMJ performs microextraction within a well-defined area of the tissue using the liquid microjunction interface (303–305). The principle of LMJ is present in **Figure 3D**. In brief, a probe aspirates a certain amount of extraction solvent and dispenses a portion onto the tissue surface to create a liquid microjunction between the probe and the tissue surface. Analytes that are soluble in the solvent will be extracted into the liquid microjunction. After a predefined extraction time, the probe is aspirated and the extraction solution can be directly dispensed to LC-MS system (163, 306). Alternatively, it is possible to perform several cycles of extraction and pool all the collected solution in the same vial to increase the quantity of samples for the further analysis (307).

For spatially resolved microproteomics, there are two LMJ strategies (308). Firstly, localized on-tissue digestion is performed and digested peptides are extracted by LMJ (293). Secondly, intact proteins are directly extracted from regions of interest within the tissue (309). In conclusion, LMJ has shown great capacities in extraction of proteins from specific cell subpopulation, contributing extensively to the proteomic analysis (310–312).

#### 3.1.3 Comparison of laser microdissection and liquid microjunction

There are some similarities between LMD and LMJ. Before being used, both of them need the histology structure of the samples, which can be obtained from histology staining, immunochemistry and MSI molecular histology images. Both of them are appliable to fresh frozen tissues, FFPE tissues and cytologic smears. There are also a few differences between them. For LMD, it can dissect regions of any size and any shape from the sample surface. It can cut off an area of tissue with a few square milimeters. It can also allow to obtain a region with a few micrometers and even submicrometers in diameter. Therefore, LMD can isolate a large area of tissue, cell clusters, single cell and subcellular compartments. For LMJ, the droplet deposited on the sample surface is a circle 0.25 to 4 mm in diameter (313). LMJ is more appropriate to sample with the larger surface area (314). Besides, the dissected sample obtained by LMD needs to be extracted and it is always challenging to process the small volume of sample. LMJ can perform the extraction *in situ* from the target surface area of the sample and the extracts can be directly introduced into the LC-MS system. During the process, the sample consumption is largely reduced.

### 3.2 Application of spatially resolved microproteomics in endocrine tumors

LMD or LMJ coupled with MS/MS makes full use of their advantages in the analysis of heterogenous endocrine tumor tissues, allowing for in-depth proteomic analysis and capable of depicting the underlying protein alternations in the endocrine tumor microenvironment (315, 316).

Prolactinoma is a subtype of pituitary adenoma and encompasses various types of cells including prolactin cells, endothelial cells, fibroblasts and other stomal cells (317). To better explain the prolactinoma tumorigenesis from the proteomics level, Liu et al. dissected pure prolactin cells from prolactinomas using immune-LMD and performed bottom-up proteomic analysis on the extracted proteins (318). By searching the human International Protein Index database with MS/MS spectra, they successfully set up a specific prolactinoma spectral library of 2,243 proteins.

Amyloids are abnormal proteins, which deposit in the organs and tissues, such as brain, heart, bladder, skin, thyroid, parathyroid, muscles and nerves (319–321). As the amyloid deposition increases, the normal function of organs and tissues is disturbed (322). Some types of amyloidosis are associated with the occurrence and development of the diseases (323–325). Parathyroid hormone (PTH), a polypeptide hormone, has been shown to form amyloid and amyloid-like beta-sheet aggregation in parathyroid adenomas (326). Colombat et al. used LMD-LC-MS/MS to analyze the protein profiling for parathyroid adenomas whose histological analysis presented nodular typical amyloid deposits. And the LMD-LC-MS/MS spectra successfully identified the amyloid fibril protein in parathyroid adenomas as PTH (327). They speculated that the formation of amyloid in a subset of parathyroid adenomas resulted from inappropriate PTH production. The physiological hormone aggregation might escape the control of functional amyloid processes, leading to disease-amyloid aggregation of PTH.

The pituitary gland can be divided into two distinct regions both in anatomy and function: the anterior pituitary (adenohypophysis, AH) and the posterior pituitary (neurohypophysis, NH). The anterior pituitary comprises of five different types of epithelial endocrine cells, responsible for secreting GH, PRL, TSH, ACTH, follicle stimulating hormone and luteinizing hormone (328). The posterior secrets two hormones: oxytocin and vasopressin. Kertesz et al. used an automated LMJ system for profiling of arginine vasopressin and ACTH in normal human pituitary gland and pituitary adenomas (329). This spatially resolved sampling approach allowed selective protein extraction from the anterior and the posterior regions of the human pituitary gland as well as selective protein extraction from the tumor region and the normal posterior region of the ACTH secreting adenoma tissue. The separation and identification of the extracted proteins were processed with LC-MS/MS system. Heatmaps were created to show that arginine vasopressin was mostly distributed in NH regions and ACTH in AH regions. ACTH levels in secreting adenomas and normal AH regions were significantly higher than in non-secreting adenomas and NH regions. The results showed that the signature of arginine vasopressin and ACTH in a series of ACTH secreting and non-secreting pituitary adenomas was consistent with the histopathological evaluation.

## 4 Conclusion and perspective

Multi-omics analysis for endocrine tumors is gaining much attention in recent years (330–332). Endocrine tumors are characterized by a marked diversity and high heterogeneity. Most endocrine tumors are benign, evolving locally and slowly. However, a fraction of endocrine tumors are malignant, as evidenced by metastasis and fatal evolution (2). Biomarkers associated with tumorigenesis, progression and metastasis are intensively investigated, facilitating the development of novel diagnostic tools and promising treatments. MSI techniques show the strength in detection and identification of proteins, lipids and metabolites that altered significantly between the tumor tissue and the normal tissue. Compared with non-metastatic PTC, thioredoxin, S100-A10 and S100-A6 were significantly elevated in metastatic PTC (*p* values < 0.005). And the three proteins were identified as protein biomarkers for PTC with lymph node metastasis. Besides, CL species with an abnormal abundance and diversity are identified as candidate biomarkers for oncocytic thyroid tumor, such as CL (72:6), CL (72:8) and CL (76:9). Moreover, MSI result showed aldosterone and 18-oxocortisol congregated within the tumor regions where aldosterone synthase was distributed, serving as a complementary for the view that aldosterone or 18-oxocortisol has the potential to act as a biomarker for APA. With the advances in LC-MS and microextraction approaches, spatially resolved microproteomics in endocrine tumors has exhibited excellent performances in revealing the regional protein profiles within the heterogeneous tumor tissues.

The samples for endocrine tumors mainly comprise of fresh-frozen tissues, FFPE tissues and cytologic samples. Proteomics, lipidomics and metabolomics guided by MSI and spatially resolved microproteomics can reflect the relative abundance and spatial distribution of analytes. The sample preparation protocols are crucial and need to be established based on the purpose of the study and the collected samples. It should be taken into account to protect the analytes from degradation and displacement within the tissue and preserve the integrity of the tissue during the preparation process.

MSI provides spatially resolved molecular analysis of biological samples without labelling. However, MSI is disadvantageous in molecule identification caused by local ion suppression and has limitations in the depth of molecule detection coverage compared with established proteomics, lipidomics and metabolomics based on LC-MS/MS analysis (333). The strategy that combines MSI, microextraction approaches and LC-MS has the potential to solve the above problem. In brief, the tissue is first analyzed with MSI to produce localization-registered mass spectra and ion images. The tissue is then segmented into different regions. And, the location information of the target region is passed to the LMD or LMJ. The microextraction is performed on the target regions. Lastly, the extracts are analyzed with LC-MS/MS. This strategy allows more comprehensive and deeper insights into the molecular heterogeneity uncovered by MSI and enables a better understanding of the molecular mechanism within the sample (289). It has shown the potential of improving the characterization and identification of proteins associated with endocrine tumors (329). One limitation for this strategy in lipidomcis and metabolomics is the small sample quantity obtained by microextraction, which poses challenges to the following LC-MS/MS analysis. Therefore, mass spectrometers and chromatographic methods with significantly enhanced sensitivity are required in this filed.

Advanced MSI techniques are remarkably promising in single cell metabolomics, where the analysis on metabolites is directly performed on single cells without any cell lysis, separation or label (334). The spatial resolution of SIMS-MSI with GCIBs as the primary ion beam can approach 1 μm, capable of imaging a single cell (335). A spatial resolution of around 1.4 μm has been achieved by the development of atmospheric pressure MALDI MSI platform (336). High spatial resolution MALDI-MSI (down to 0.5-5 μm) using both reflection and transmission geometries has been being developed by the Caprioli group (337, 338). MALDI-MSI is capable of mapping and visualizing lipids in a single cell of newly fertilized individual zebrafish embryos (339). MALDI-2 is a post post-ionization technique. After the initial MALDI ionization, a second laser that is parallel to the sample surface is applied to post-ionize neutral molecules. MALDI-2 reduces ion suppression effects and improves sensitivity by up to 3 orders of magnitude. And spatial resolution can reach 5 μm. By applying transmission-mode MALDI-2 ion source in MSI of the brain tissue, the subcellular resolution was achieved (340). MSI-based single cell metabolomics devotes to profiling metabolites spatially and/or temporally in a single cell level, providing insights into the intracellular and intercellular metabolic activities and revealing the intercellular heterogeneity.

With the development in mass spectrometry, chromatography, microextraction methods, sample preparation protocols and data analysis methods, analyses on the proteomics, lipidomics and metabolomics of endocrine tumors will provide new dimensional insights in molecular level, cellular even subcellular level and tissue level, aiding in overcoming the problems of pathophysiology, diagnosis, and treatment for endocrine tumors.

## Author contributions

XZ and YH conceived of the concept and idea of this article. YH collected references, wrote and revised the manuscript, and was responsible for the corresponding work of the manuscript. SG collected partial references. YG, ZZ, RC and XZ participated in proofreading, editing and revision of the manuscript. All authors contributed to the article and approved the submitted version.

## Glossary

**Table:**

ACTH	adrenocorticotropic hormone
AH	adenohypophysis
AIP	aryl hydrocarbon receptor interacting protein
APA	aldosterone-producing adenoma
APCC	aldosterone-producing cell cluster
ATC	anaplastic thyroid carcinoma
BRAF	v-Raf murine sarcoma viral oncogene homolog B1
CACNA1D	calcium voltage-gated channel subunit alpha1 D
CHCA	alpha-cyano-4-hydroxycinnamic acid
CLs	cardiolipins
DESI	desorption electrospray ionization
DESI-MSI	desorption electrospray ionization-mass spectrometry imaging
2,5-DHB	2,5-dihydroxybenzoic acid
ESI	electrospray ionization
FFAs	free fatty acids
FFPE	formalin fixed paraffin embedded
FNA	fine needle aspiration
GCIBs	gas cluster ion beams
GH	growth hormone
GNAS	guanine nucleotide binding protein, alpha stimulating
IHC	immunohistochemistry
IR	infrared
ITO	indium-tin oxide
KCNJ5	potassium voltage-gated channel subfamily J member 5
LMD	laser microdissection
LC-MS	liquid chromatography-mass spectrometry
LC-MS/MS	liquid chromatography with tandem mass spectrometry
LDI	laser desorption/ionization
LMJ	liquid microjunction
LMIGs	liquid metal ion guns
*m/z*	mass-to-charge ratio
MALDI	matrix-assisted laser desorption/ionization
MALDI-MSI	matrix-assisted laser desorption/ionization-mass spectrometry imaging
MEN1	menin 1
MS	mass spectrometry
MSI	mass spectrometry imaging
MTC	medullary thyroid carcinoma
NH	neurohypophysis
PA	phosphatidic acid
PC	phosphatidylcholine
PEN	polyethylene napthalate
PLs	phospholipids
PTC	papillary thyroid carcinoma
PTH	parathyroid hormone
PRL	prolactin
RAS	rapidly accelerated fibrosarcoma
RET	Proto-oncogene tyrosine-protein kinase receptor Ret
SA	sinapinic acid
SCD1	stearoyl-CoA desaturase
SIMS	secondary ion mass spectrometry
SIMS-MSI	secondary ion mass spectrometry-mass spectrometry imaging
SM	sphingomyelin
S100-A10 p11	the ligand of Annexin-II
S100-A6	Calcyclin
MS/MS	Tandem mass spectrometer
TOF	time-of-flight
TSH	thyroid stimulating hormone
UV	ultraviolet
