# Supplementary material for: Applications of spatially resolved omics in the field of endocrine tumors
Source: Front Endocrinol (Lausanne). 2023 Jan 10;13:993081. doi: 10.3389/fendo.2022.993081 (PMC9873308; doi:10.3389/fendo.2022.993081)
Supplement: Supplementary file 1 [file Table_1.docx]

Supplementary Material

Supplementary Table 1. Summary of spatially resolved proteomics, lipidomics, and metabolomics on endocrine tumors on endocrine tumors

| Target molecules | Sample type | Endocrine tumor type | Method | Reference |
| --- | --- | --- | --- | --- |
| Proteins | Cytologic samples | Thyroid carcinoma | MALDI-MSI | (1) |
| Proteins | Cytologic samples | Malignant thyroid nodules | MALDI-MSI | (2) |
| Proteins | Cytologic samples | Benign, intermediate and malignant thyroid nodules | MALDI-MSI | (3) |
| Proteins | Cytologic samples | Thyroid nodules | MALDI-MSI | (4) |
| Proteins | Cytological samples | PTC and benign thyroid nodules | MALDI-MSI | (5) |
| Proteins | Cytologic samples | PTC, Hp, FA, and HT | MALDI-MSI | (6) |
| Proteins | Cytologic samples | cv-PTC, fv-PTC, Hp, HCA, MG and MTC | MALDI-MSI | (7) |
| Proteins | Cytologic samples | Hp, cv-PTC, fv-PTC and uncertain malignant potential tumors | MALDI-MSI | (8) |
| Proteins | Cytologic samples | Noninvasive follicular thyroid neoplasms with papillary-like nuclear features and HCA | MALDI-MSI | (9) |
| Proteins | Cytological samples | Hp, HT and PTC | MALDI-MSI | (10) |
| Proteins | Fresh frozen tissues | PTC | MALDI-MSI | (11) |
| Proteins | Fresh frozen tissues | PTC | MALDI-MSI | (12) |
| Proteins | Fresh frozen tissues | Pituitary adenoma | MALDI-MSI | (13) |
| Proteins | Fresh frozen tissues | Prolactinoma | LMD and LC-MS/MS | (14) |
| Proteins | Fresh frozen tissues | Prolactinoma | LMD and LC-MS/MS | (15) |

Supplementary Table 1. *Cont.*

| Target molecules | Sample type | Endocrine tumor type | Method | Reference |
| --- | --- | --- | --- | --- |
| Proteins | Fresh frozen tissues | Pituitary adenoma | MALDI-MSI, LMJ and LC-MS | (16) |
| Proteins | FFPE tissues and fresh frozen tissues | PTC | MALDI-MSI | (17) |
| Proteins | FFPE tissues | Parathyroid adenoma | LMD and LC-MS | (18) |
| Proteins | FFPE tissues | cv-PTC, fv-PTC and noninvasive follicular thyroid neoplasms with papillary-like nuclear features | MALDI-MSI | (19, 20) |
| Proteins | FFPE tissues | Oncocytic follicular thyroid carcinoma | MALDI-MSI | (21) |
| Proteins | FFPE tissues | Hp, FA, cv-PTC, fv-PTC and uncertain malignant potential tumors | MALDI-MSI | (22) |
| Proteins | FFPE tissues | cv-PTC, fv-PTC, MTC, FA and ATC | MALDI-MSI | (23) |
| Proteins | FFPE tissues | cv-PTC, fv-PTC, FTC, MTC and ATC | MALDI-MSI | (24) |
| Proteins | FFPE tissues | MTC | MALDI-MSI | (25) |
| Proteins | FFPE tissues | PTC | MALDI-MSI | (26) |
| Proteins |  | PTC | MALDI-MSI | (27) |
| Lipids | Fresh frozen tissues | PTC and FTC | MALDI-MSI | (28) |
| Lipids | Fresh frozen tissues | MG, MTC and benign thyroid adenoma | MALDI-MSI | (29) |
| Lipids | Fresh frozen tissues | PTC | MALDI-MSI | (30) |
| Lipids | Fresh frozen tissues | PTC | MALDI-MSI | (31) |
| Lipids | Fresh frozen tissues | HCA, PTC, FTC and Hürthle cell carcinoma | DESI-MSI | (32) |
| PC | Cytologic samples | ATC | SIMS-MSI | (33) |

Supplementary Table 1. *Cont.*

| Target molecules | Sample type | Endocrine tumor | Method | Reference |
| --- | --- | --- | --- | --- |
| FFA and PL | Fresh frozen tissues | Thyroid carcinoma | MALDI-MSI | (34) |
| Steroids | Fresh frozen tissues | Pheochromocytoma | MALDI-MSI | (35) |
| Steroids | Fresh frozen tissues | APA and APCC | MALDI-MSI | (36) |
| Steroids | FFPE tissues | ACC | MALDI-MSI | (37) |
| Lipids and metabolites | Fresh frozen tissues | Oncocytic thyroid tumors | DESI, LMD and LC-MS | (38) |
| Lipids and Metabolites | Fresh frozen tissues | PTC | DESI-MSI | (39) |
| Metabolites | Cytologic samples | HCA, FA, FTC, PTC and Hürthle cell carcinoma | DESI-MSI | (40) |
| Metabolites | Fresh frozen tissues | PTC | DESI-MSI | (41) |
| Metabolites | FFPE tissues | Thyroid carcinoma | MALDI-MSI | (42) |
| Metabolites | FFPE tissues | Paraganglioma | MALDI-MSI | (43) |
| Metabolites | FFPE tissues | APA and APCC | MALDI-MSI | (44) |
| Metabolites | FFPE tissues | APA | MALDI-MSI | (45) |
| Glycans | FFPE tissues | Thyroid carcinoma | MALDI-MSI | (46) |
| Glycans | FFPE tissues | Thyroid tumor | MALDI-MSI | (47) |

ACC: adrenocortical carcinoma; APA: aldosterone-producing adenoma; APCC: aldosterone-producing cell cluster; ATC: anaplastic thyroid carcinoma; cv-PTC: conventional variant-papillary thyroid carcinoma; DESI: desorption electrospray ionization; DESI-MSI: desorption electrospray ionization-mass spectrometry imaging; FA: follicular adenoma; FFA: free fatty acid; FFPE: formalin fixed paraffin embedded; FTC: follicular thyroid carcinoma; fv-PTC: follicular variant-papillary thyroid cancer; HCA: Hürthle cell carcinoma; Hp: hyperplastic nodules; HT: Hashimoto’s thyroiditis; LC-MS: liquid chromatography-mass spectrometry; LMD: laser microdissection; LMJ: liquid microjunction; MALDI-MSI: matrix-assisted laser desorption/ionization-mass spectrometry imaging; MG: multinodular goitre; MTC: medullary thyroid carcinoma; PC: phosphatidylcholine; PL: phospholipid; PTC: papillary thyroid carcinoma; SIMS-MSI: secondary ion mass spectrometry-mass spectrometry imaging.

# Reference

1. Mosele N, Smith A, Galli M, Pagni F, Magni F. MALDI-MSI Analysis of Cytological Smears: The Study of Thyroid Cancer. Methods in molecular biology (Clifton, NJ). 2017;1618:37-47.

2. Piga I, Capitoli G, Clerici F, Mahajneh A, Brambilla V, Smith A, et al. Ex vivo thyroid fine needle aspirations as an alternative for MALDI-MSI proteomic investigation: intra-patient comparison. Analytical and Bioanalytical Chemistry. 2021;413(5):1259-66.

3. Piga I, Capitoli G, Tettamanti S, Denti V, Smith A, Chinello C, et al. Feasibility Study for the MALDI-MSI Analysis of Thyroid Fine Needle Aspiration Biopsies: Evaluating the Morphological and Proteomic Stability Over Time. Proteomics Clinical Applications. 2019;13(1).

4. Piga I, Capitoli G, Denti V, Tettamanti S, Smith A, Stella M, et al. The management of haemoglobin interference for the MALDI-MSI proteomics analysis of thyroid fine needle aspiration biopsies. Analytical and Bioanalytical Chemistry. 2019;411(20):5007-12.

5. Galli M, Zoppis I, De Sio G, Chinello C, Pagni F, Magni F, et al. A Support Vector Machine Classification of Thyroid Bioptic Specimens Using MALDI-MSI Data. Advances in bioinformatics. 2016;2016:3791214-.

6. Pagni F, De Sio G, Garancini M, Scardilli M, Chinello C, Smith AJ, et al. Proteomics in thyroid cytopathology: Relevance of MALDI-imaging in distinguishing malignant from benign lesions. Proteomics. 2016;16(11-12):1775-84.

7. Pagni F, Mainini V, Garancini M, Bono F, Vanzati A, Giardini V, et al. Proteomics for the diagnosis of thyroid lesions: preliminary report. Cytopathology. 2015;26(5):318-24.

8. Mainini V, Pagni F, Garancini M, Giardini V, De Sio G, Cusi C, et al. An Alternative Approach in Endocrine Pathology Research: MALDI-IMS in Papillary Thyroid Carcinoma. Endocr Pathol. 2013;24(4):250-3.

9. Piga I, Capitoli G, Clerici F, Brambilla V, Leni D, Scardilli M, et al. Molecular trait of follicular-patterned thyroid neoplasms defined by MALDI-imaging. Biochimica Et Biophysica Acta-Proteins and Proteomics. 2020;1868(11).

10. Capitoli G, Piga I, Clerici F, Brambilla V, Mahajneh A, Leni D, et al. Analysis of Hashimoto's thyroiditis on fine needle aspiration samples by MALDI-Imaging. Biochim Biophys Acta Proteins Proteom. 2020;1868(11):140481.

11. Min K-W, Bang J-Y, Kim KP, Kim W-S, Lee SH, Shanta SR, et al. Imaging Mass Spectrometry in Papillary Thyroid Carcinoma for the Identification and Validation of Biomarker Proteins. Journal of Korean Medical Science. 2014;29(7):934-+.

12. Meding S, Nitsche U, Balluff B, Elsner M, Rauser S, Schoene C, et al. Tumor Classification of Six Common Cancer Types Based on Proteomic Profiling by MALDI Imaging. Journal of Proteome Research. 2012;11(3):1996-2003.

13. Calligaris D, Feldman DR, Norton I, Olubiyi O, Changelian AN, Machaidze R, et al. MALDI mass spectrometry imaging analysis of pituitary adenomas for near-real-time tumor delineation. Proceedings of the National Academy of Sciences of the United States of America. 2015;112(32):9978-83.

14. Liu YC, Zhuang DX, Hou RP, Li JA, Xu GM, Song T, et al. Shotgun proteomic analysis of microdissected postmortem human pituitary using complementary two-dimensional liquid chromatography coupled with tandem mass spectrometer. Anal Chim Acta. 2011;688(2):183-90.

15. Liu YC, Wu JS, Yan GQ, Hou RP, Zhuang DX, Chen LP, et al. Proteomic analysis of prolactinoma cells by immuno-laser capture microdissection combined with online two-dimensional nano-scale liquid chromatography/mass spectrometry. Proteome Sci. 2010;8.

16. Kertesz V, Calligaris D, Feldman DR, Changelian A, Laws ER, Santagata S, et al. Profiling of adrenocorticotropic hormone and arginine vasopressin in human pituitary gland and tumor thin tissue sections using droplet-based liquid-microjunction surface-sampling-HPLC-ESI-MS-MS. Analytical and Bioanalytical Chemistry. 2015;407(20):5989-98.

17. Nipp M, Elsner M, Balluff B, Meding S, Sarioglu H, Ueffing M, et al. S100-A10, thioredoxin, and S100-A6 as biomarkers of papillary thyroid carcinoma with lymph node metastasis identified by MALDI Imaging. Journal of Molecular Medicine-Jmm. 2012;90(2):163-74.

18. Colombat M, Barres B, Renaud C, Ribes D, Pericard S, Camus M, et al. Mass spectrometry-based proteomic analysis of parathyroid adenomas reveals PTH as a new human hormone-derived amyloid fibril protein. Amyloid. 2021;28(3):153-7.

19. Ucal Y, Tokat F, Duren M, Ince U, Ozpinar A. Investigating the peptide profile of noninvasive follicular thyroid neoplasm with papillary-like nuclear features (NIFTP): application of MALDI mass spectrometry imaging. Febs Open Bio. 2019;9:402-3.

20. Ucal Y, Tokat F, Duren M, Ince U, Ozpinar A. Peptide Profile Differences of Noninvasive Follicular Thyroid Neoplasm with Papillary-Like Nuclear Features, Encapsulated Follicular Variant, and Classical Papillary Thyroid Carcinoma: An Application of Matrix-Assisted Laser Desorption/Ionization Mass Spectrometry Imaging. Thyroid. 2019;29(8):1125-37.

21. Abdelmoula WM, Skraskova K, Balluff B, Carreira RJ, Tolner EA, Lelieveldt BPF, et al. Automatic Generic Registration of Mass Spectrometry Imaging Data to Histology Using Nonlinear Stochastic Embedding. Analytical Chemistry. 2014;86(18):9204-11.

22. Galli M, Pagni F, De Sio G, Smith A, Chinello C, Stella M, et al. Proteomic profiles of thyroid tumors by mass spectrometry-imaging on tissue microarrays. Biochimica Et Biophysica Acta-Proteins and Proteomics. 2017;1865(7):817-27.

23. Kurczyk A, Gawin M, Chekan M, Wilk A, Lakomiec K, Mrukwa G, et al. Classification of Thyroid Tumors Based on Mass Spectrometry Imaging of Tissue Microarrays; a Single-Pixel Approach. International Journal of Molecular Sciences. 2020;21(17).

24. Pietrowska M, Diehl HC, Mrukwa G, Kalinowska-Herok M, Gawin M, Chekan M, et al. Molecular profiles of thyroid cancer subtypes: Classification based on features of tissue revealed by mass spectrometry imaging. Biochimica Et Biophysica Acta-Proteins and Proteomics. 2017;1865(7):837-45.

25. Smith A, Galli M, Piga I, Denti V, Stella M, Chinello C, et al. Molecular signatures of medullary thyroid carcinoma by matrix-assisted laser desorption/ionisation mass spectrometry imaging. Journal of Proteomics. 2019;191:114-23.

26. Gawin M, Kurczyk A, Stobiecka E, Fratczak K, Polanska J, Pietrowska M, et al. Molecular Heterogeneity of Papillary Thyroid Cancer: Comparison of Primary Tumors and Synchronous Metastases in Regional Lymph Nodes by Mass Spectrometry Imaging. Endocr Pathol. 2019;30(4):250-61.

27. Mueller M, Heiliger K-J, Rauser S, Meding S, Balluf B, Braselmann H, et al. MALDI-TOF IMS REVEALS A DIFFERENT PROTEIN EXPRESSION PROFILE FOR PTC WITH AND WITHOUT METASTASIS. Cellular Oncology. 2009;31(2):118-9.

28. Guo S, Wang YM, Zhou D, Li ZL. Significantly increased monounsaturated lipids relative to polyunsaturated lipids in six types of cancer microenvironment are observed by mass spectrometry imaging. Sci Rep-Uk. 2014;4.

29. Guo S, Qiu L, Wang Y, Qin X, Liu H, He M, et al. Tissue imaging and serum lipidomic profiling for screening potential biomarkers of thyroid tumors by matrix-assisted laser desorption/ionization-Fourier transform ion cyclotron resonance mass spectrometry. Analytical and Bioanalytical Chemistry. 2014;406(18):4357-70.

30. Ishikawa S, Tateya I, Hayasaka T, Masaki N, Takizawa Y, Ohno S, et al. Increased expression of phosphatidylcholine (16:0/18:1) and (16:0/18:2) in thyroid papillary cancer. PLoS One. 2012;7(11):e48873.

31. Wojakowska A, Cole LM, Chekan M, Bednarczyk K, Maksymiak M, Oczko-Wojciechowska M, et al. Discrimination of papillary thyroid cancer from non-cancerous thyroid tissue based on lipid profiling by mass spectrometry imaging. Endokrynologia Polska. 2018;69(1):2-8.

32. Zhang J, Yu W, Ryu SW, Lin J, Buentello G, Tibshirani R, et al. Cardiolipins Are Biomarkers of Mitochondria-Rich Thyroid Oncocytic Tumors. Cancer Research. 2016;76(22):6588-97.

33. Nygren H, Hagenhoff B, Malmberg P, Nilsson M, Richter K. Bioimaging TOF-SIMS: High resolution 3D Imaging of single cells. Microscopy Research and Technique. 2007;70(11):969-74.

34. Wang S-S, Wang Y-J, Zhang J, Sun T-Q, Guo Y-L. Derivatization Strategy for Simultaneous Molecular Imaging of Phospholipids and Low-Abundance Free Fatty Acids in Thyroid Cancer Tissue Sections. Analytical Chemistry. 2019;91(6):4070-6.

35. Takeo E, Sugiura Y, Uemura T, Nishimoto K, Yasuda M, Sugiyama E, et al. Tandem Mass Spectrometry Imaging Reveals Distinct Accumulation Patterns of Steroid Structural Isomers in Human Adrenal Glands. Anal Chem. 2019;91(14):8918-25.

36. Sugiura Y, Takeo E, Shimma S, Yokota M, Higashi T, Seki T, et al. Aldosterone and 18-Oxocortisol Coaccumulation in Aldosterone-Producing Lesions. Hypertension. 2018;72(6):1345-54.

37. Sun N, Kunzke T, Sbiera S, Kircher S, Feuchtinger A, Aichler M, et al. Prognostic Relevance of Steroid Sulfation in Adrenocortical Carcinoma Revealed by Molecular Phenotyping Using High-Resolution Mass Spectrometry Imaging. Clin Chem. 2019;65(10):1276-86.

38. Feider CL, Elizondo N, Eberlin LS. Ambient Ionization and FAIMS Mass Spectrometry for Enhanced Imaging of Multiply Charged Molecular Ions in Biological Tissues. Analytical Chemistry. 2016;88(23):11533-41.

39. Zhang JL, Feider CL, Nagi C, Yu WD, Carter SA, Suliburk J, et al. Detection of Metastatic Breast and Thyroid Cancer in Lymph Nodes by Desorption Electrospray Ionization Mass Spectrometry Imaging. J Am Soc Mass Spectr. 2017;28(6):1166-74.

40. DeHoog RJ, Zhang J, Alore E, Lin JQ, Yu W, Woody S, et al. Preoperative metabolic classification of thyroid nodules using mass spectrometry imaging of fine-needle aspiration biopsies. Proceedings of the National Academy of Sciences of the United States of America. 2019;116(43):21401-8.

41. Huang LJ, Mao XX, Sun CL, Luo ZG, Song XW, Li X, et al. A graphical data processing pipeline for mass spectrometry imaging-based spatially resolved metabolomics on tumor heterogeneity. Anal Chim Acta. 2019;1077:183-90.

42. Ucal Y, Ozpinar A. Improved spectra for MALDI MSI of peptides using ammonium phosphate monobasic in MALDI matrix. Journal of Mass Spectrometry. 2018;53(8):635-48.

43. Murakami M, Sun N, Greunke C, Feuchtinger A, Kircher S, Deutschbein T, et al. Mass spectrometry imaging identifies metabolic patterns associated with malignant potential in pheochromocytoma and paraganglioma. Eur J Endocrinol. 2021;185(1):179-91.

44. Sun N, Meyer LS, Feuchtinger A, Kunzke T, Knosel T, Reincke M, et al. Mass Spectrometry Imaging Establishes 2 Distinct Metabolic Phenotypes of Aldosterone-Producing Cell Clusters in Primary Aldosteronism. Hypertension. 2020;75(3):634-44.

45. Murakami M, Rhayem Y, Kunzke T, Sun N, Feuchtinger A, Ludwig P, et al. In situ metabolomics of aldosterone-producing adenomas. Jci Insight. 2019;4(17).

46. Scott DA, Norris-Caneda K, Spruill L, Bruner E, Kono Y, Angel PM, et al. Specific N-linked glycosylation patterns in areas of necrosis in tumor tissues. International Journal of Mass Spectrometry. 2019;437:69-76.

47. Drake RR, Talbot K, Norris-Caneda K, Bruner E, Angel PM. Comprehensive spatial mapping by MALDI imaging mass spectrometry of the N-glycan tissue glycome and database generation for eighteen tumor types. Cancer Research. 2017;77.
